# Behavioral and Brain Activity Indices of Cognitive Control Deficits in Binge Drinkers

**DOI:** 10.3390/brainsci8010009

**Published:** 2018-01-04

**Authors:** Sean M. Molnar, Lauren E. Beaton, Joseph P. Happer, Lee A. Holcomb, Siyuan Huang, Donatello Arienzo, Ksenija Marinkovic

**Affiliations:** 1 Spatio-Temporal Brain Imaging Lab, Department of Psychology, San Diego State University, San Diego, CA 92120, USA; sean.michael.molnar@gmail.com (S.M.M.); lbeaton@mail.sdsu.edu (L.E.B.); jhapper@sdsu.edu (J.P.H.); leeaholcomb@gmail.com (L.A.H.); syhuang.thu@gmail.com (S.H.); darienzo@sdsu.edu (D.A.); 2 Department of Radiology, University of California, San Diego, CA 92039, USA

**Keywords:** binge drinking, response conflict, Stroop, ventrolateral prefrontal cortex, thalamus

## Abstract

Heavy episodic drinking is prevalent among young adults and is a public issue of increasing importance. Its initiation and maintenance are associated with deficits in the capacity to inhibit automatic processing in favor of non-habitual responses. This study used functional magnetic resonance imaging (fMRI) to examine behavioral and brain activity indices of cognitive control during the Stroop task as a function of binge drinking. Heavy episodic drinkers (HED) reported consuming 5+/6+ drinks in two hours at least five times in the past six months and were compared to light drinkers (LED) who reported two or fewer binge episodes but were matched on demographics, intelligence and family history of alcoholism. Greater conflict-induced activity in the ventrolateral prefrontal cortex (VLPFC) and thalamus was observed in HED participants and it was positively correlated with alcohol intake and alcohol-related harmful consequences. HEDs maintained intact accuracy but at a cost of prolonged reaction times to high-conflict trials and increased ratings of task difficulty. Greater activation of the areas implicated in cognitive control is consistent with compensatory network expansion to meet higher cognitive demands. These results provide further insight into degradation of cognitive control in HEDs which may benefit development of detection and prevention strategies.

## 1. Introduction

Cognitive control is a facet of executive functioning that underlies optimization of behavior by integrating appropriate response selection with previous experiences and current goals [1,2,3,4,5]. These top-down influences have been explored with tasks that probe decision making under conditions that induce response interference and selective inhibition [6,7,8]. Extensive neuroimaging literature has characterized the predominantly frontal cortical network comprised of the anterior cingulate, ventrolateral (VLPFC) and dorsolateral prefrontal cortices (DLPFC), insula, as well as the parietal cortex and basal ganglia [6,9,10,11,12,13,14,15,16]. The neurofunctional system subserving cognitive control is particularly vulnerable to the effects of both acute alcohol intoxication [17,18,19] and long-term excessive alcohol use [20,21,22,23]. Structural imaging studies indicate that alcohol use disorder (AUD) is associated with compromised white matter tracts, reductions in hippocampal volume, and decreases in cortical thickness [24,25,26,27,28,29]. These measures are predictive of clinical outcomes such as the duration of abstinence after treatment [30]. Results of functional magnetic resonance imaging (fMRI) studies using executive tasks are less consistent with some studies showing decreased [31,32,33], and others reporting increased activation in participants with AUD compared with controls [34,35,36,37,38,39,40]. Activation increase in AUD participants is commonly observed in the absence of executive task performance deficits suggesting that compensatory mechanisms are engaged to maintain normative behavioral performance especially on tasks imposing higher cognitive demands.

Heavy episodic drinking (HED), also termed binge drinking, is a variation of alcohol use characterized by high volume drinking episodes interspersed with periods of low or no intake. It has been on the rise especially among young and emerging adults, raising serious concerns about the neurotoxic effects of alcohol on a large scale [41]. Neurobiologically based accounts of addiction conceptualize HED as a stage in an addiction cycle comprised of withdrawal periods followed by active alcohol seeking/craving behaviors [42,43,44]. While acute alcohol-induced pharmacological effects result in enhanced neural inhibition overall [45,46], protracted periods of heavy episodic drinking elicit neuroadaptive changes to compensate for alcohol’s effects on the brain [42,47]. This is reflected in neural hyperexcitability and associated with symptoms of withdrawal, dysphoria and increased risk of relapse and dependence [48,49,50,51]. Evidence is accumulating in support of the “continuum hypothesis” where HED may serve as a precursor to AUD [52,53,54]. Structural imaging studies have shown that the effects of HED are particularly deleterious during critical stages of brain development and frontal lobe maturation in adolescence and emerging young adulthood [54,55,56,57]. Furthermore, HED is associated with impaired executive functions [58,59,60,61]. Neuroimaging studies of cognitive control functions, however, have reported mixed results with HED participants showing decreased fMRI activation on response inhibition [33,62]. Conversely, increased activation during response inhibition was observed in heavy drinking adolescents [63]. Increased activation in frontal regions was reported with greater reliability during more complex tasks engaging executive functions such as spatial interference [64] and working memory [65]. These findings have been interpreted as reflecting compensatory engagement of cortical areas needed to maintain performance accuracy during cognitively demanding tasks. They primarily encompass frontal regions suggesting an underlying dysregulation of cognitive control which is an important factor in the development of AUD. Indeed, deficient self-regulation can contribute to the formation of habitual, compulsive alcohol consumption resulting in impaired capacity to refrain from drinking [66,67,68,69,70,71,72].

Despite the prevalence of HED and its importance for public health, there is a paucity of studies on the associated harmful consequences in the neurocognitive domain. Most studies report no deficits in intelligence or on task performance in HED indicating that more sensitive neuroimaging measures are needed to characterize deficits associated with binge drinking patterns [54,73,74]. Given the importance of cognitive control for the development of AUD, the aim of the present study was to examine its neural underpinnings and possible emerging signs of excessive drinking habits in HED. Cognitive control is commonly probed with tasks that involve inhibition of automatic responses in favor of those that are task-relevant but non-habitual [75,76]. Participants performed a version of the color-Stroop response conflict task, which has been shown to elicit conflict interference with high sensitivity to alcohol intoxication effects [17,19], during functional magnetic resonance imaging (fMRI). HED and matched light episodic drinkers (LED) were compared on behavioral and brain indices of response conflict in the context of a comprehensive set of questionnaires on alcohol-related behaviors, dispositional traits, personality, and intelligence measures.

## 2. Materials and Method

### 2.1. Research Participants

Thirty-one healthy, right-handed young adults (18 female, age 24.7 ± 3.9) participated in the study. They had no history of seizures, traumatic brain injury or concussions, no neurological or neuropsychiatric disorders, vision or hearing problems, and they all complied with MRI safety criteria. Participants were medication-free, they reported using no drugs or tobacco products for at least one month prior to the study and none had previously sought or been enrolled in alcohol abuse treatment. Based on the screening information on alcohol consumption rate, frequency and pattern, participants were assigned to heavy episodic drinking group (HED, *n* = 14) if they reported engaging in ≥5 binge episodes in the previous six months. Light episodic drinking (LED) group comprised individuals who had ≤2 binge episodes in that interval. A binge episode was defined as consuming 5+/6+ drinks for women/men within a two-hour time frame based on research evidence indicating that it is likely to reach a legal level of intoxication (0.08%) [77,78]. The two groups were matched on age, gender, education and family history of alcoholism (see Table 1 for group characteristics). The HED group scored higher on a wide range of alcohol-related variables, higher motor impulsivity [79], and disinhibition and boredom symptoms [80] (Table 1). Young adults were recruited from the San Diego area with flyers and on-line postings. All subjects gave written informed consent to experimental procedures approved by the relevant Institutional Review Boards. Participants were provided monetary compensation for their participation.

### 2.2. Experimental Protocol

All participants provided information on multiple dimensions of their alcohol use, such as the severity of their alcohol habit (Alcohol Use Disorder Identification Test, AUDIT) [81]; the prevalence of particular signs of alcohol misuse (Short Michigan Alcoholism Screening Test, SMAST) [82]; the number and characteristics of drinking occasions that had occurred over the past thirty days (The Time Line Follow Back, TLFB) [83]; the degree to which they crave alcohol (The Penn Alcohol Craving Scale, PACS) [84]; the reasons for engaging in drinking episodes (Drinking Motive Questionnaire Revised Short Form, DMQ-R SF) [85]; quantifying the occurrence of consequences from drinking (Brief Young Adult Alcohol Consequences Questionnaire, B-YAACQ) [86]; and personality traits (Eysenck Personality Questionnaire, EPQ) [87]. In addition, we collected information regarding the presence of depressive symptomology (Patient Health Questionnaire, EPQ) [88]; anxiety (Generalized Anxiety Disorder, GAD7) [89]; the degree of impulsive behavior for motor, attention and non-planning dimensions (Abbreviated Impulsiveness Scale, ABIS) [79]; attention deficit/hyperactivity disorder symptomology (Adult ADHD Self-Report Scale, ASRS) [90]; and finally the desire for novel situations and risk-taking behavior (Brief Measure of Sensation Seeking Scale, BSSS) [80]. Intelligence was assessed with the Wechsler Abbreviated Scale of Intelligence (WASI-II) [91]. Family history of alcoholism was assessed with a modified version of the Family History Assessment Module (FHAM) [92]. A positive family history for alcoholism (FH+) was defined as having at least one first-degree and one first- or second-degree relative, or at least three second-degree relatives diagnosed with AUD. Statistical analysis between the alcohol use measures, personality scores, and behavioral results were conducted via SPSS 24 [93]. Prior to scanning, subjects were screened for illicit substances via urinary analysis and women were tested for pregnancy and all tested negative.

### 2.3. Task

**Figure 1:**
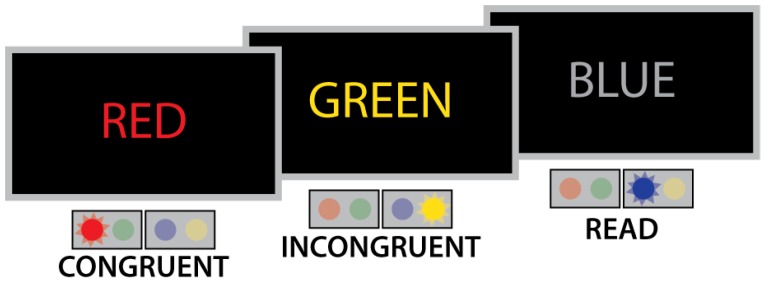
In a modified version of the Stroop task, congruent (CONG) trials consisted of color words displayed in matching colored font, incongruent (INCONG) stimuli had mismatched color words and font, and (READ) trials displayed the name of a color in a grey font. The task was to press a button corresponding to the color of the font or to the meaning of the words presented in gray. Trials were presented for 300 ms every 2 s in an optimally randomized manner.

Cognitive control processes were probed with a modified Stroop color naming task [17,19,94]. The subjects were instructed to identify the color of the font (red, green, blue or yellow) and respond as quickly and accurately as possible with index and middle fingers of both hands mapped to four buttons (Figure 1). In the congruent (CONG) condition, the color of the font matched the meaning of the color word, whereas in the incongruent (INCONG) condition the color of the font was different from the color word, inducing interference. To maintain reading dominance and automaticity, additional color words were presented in gray font and the subject responded to the meaning of the word (READ) (Figure 1). The Stroop task was presented as a randomized event-related design and consisted of 540 stimuli across four runs comprising 20% (108) CONG, 20% (108) INCONG and 60% (324) READ trials. Words were presented for a stim duration of 300 ms followed by a 1700 ms fixation (XXXX) period for a total stimulus onset asynchrony of 2 s. In addition, 108 null fixation trials were optimally interleaved with Optseq2 (http://surfer.nmr.mgh.harvard.edu/optseq/) randomization algorithm to allow for proper finite impulse response (FIR) deconvolution modeling during fMRI analysis [95]. The task was programmed with Presentation V.19.0 (Neurobehavioral Systems) to sync with the scanner through transistor–transistor (TTL) pulses.

### 2.4. Image Acquisition and Analysis

Structural and functional imaging data were collected with a GE Discovery MR750 3.0T whole body scanner equipped with an 8-channel head coil. The head was secured with a pillow and foam padding to minimize movement and maximize subject comfort. The subject was provided M3 earplugs (EAR Soft FX) to dampen scanner noise and protect hearing. A mirror was attached to the head coil to allow for comfortable viewing of the front-projected display. A high-resolution SPGR (spoiled gradient recalled echo) T1-weighted structural image sequence was acquired for each subject with the following parameters: TR = 7.38 ms, TE = 2.984 ms, flip angle = 8°, field of view (FOV) = 240, matrix 256 × 256, 170 slices, 1.2 mm slice thickness with a 94 × 94 mm in-plane resolution. During task administration, 4 runs of functional whole-brain blood oxygenation level dependent (BOLD) volumes (648 total) were collected with a T2*-weighted echo planar imaging sequence of 35 interleaved bottom-up 4 mm slices in AC-PC orientation (TR = 2000 ms, TE = 30 ms, flip angle = 90°, FOV = 220 mm, matrix 64 × 64, generating a 3.437 × 3.437 mm in-plane resolution). Anatomical and functional volumes were analyzed with AFNI (Analysis of Functional Neuroimages) 17.1.12 [96,97]. AFNI’s Montreal Neurological Institute (MNI; TT_avg152T1) template was used to warp the anatomical and functional datasets to standardized space. Volume registration of the echo planar imaging (EPI) runs was completed by setting the volume with the least number of outlier voxels as the base. A three-dimensional Gaussian kernel (FWHM 8.0) was used to blur the data within each volume and each voxel was scaled to percent signal change before deconvolution was performed. Motion correction was performed by removing TRs exceeding 0.25 mm rotational and translational motion, removing trials in which 25 percent or more of the voxels are considered outliers, and regressing out motion derivatives in deconvolution through six motion parameters and a third-order polynomial for drift. Hemodynamic response function (HRF) was modeled for each trial through AFNI’s version of finite impulse response model (also termed a “tent” function) within a time window from −4 to 12 s with respect to stimulus onset. The contrast matrix was generated by 3dDeconvolve and used for residual maximum likelihood (REML) and generalized least squares (GLSQ) statistical analysis to identify voxels with significant changes [98]. For group level statistics, the coefficients and corresponding *t*-values generated from individual REML analysis were used for mixed-effects meta-analysis (MEMA). Cluster simulations were performed via 3dClustSim at the group level to adjust for multiple comparisons and to keep *p*-values below 0.05. Region-of-interest (ROI) analysis was performed to identify the pattern of BOLD activation at anatomical locations associated with the task paradigm in order to contrast activity between heavy and light episodic drinkers. ROIs were chosen from an uncorrelated orthogonal general linear model (GLM) contrast (all conditions v. fixation periods) activation map across all subjects [99,100]. The selected anatomical locations contained active voxels clusters at a *p <* 0.0001 threshold corrected for multiple testing and family-wise error (FWE) via 3dClustSim. Beta weights representing percent signal change at each point in the time series were extracted from the ROIs for each subject. Time courses were analyzed using a mixed model ANOVA with drinking Group as a between-group factor and Condition as a within-subject factor.

## 3. Results

### 3.1. Drinking and Personality Variables

Group characteristics are displayed in Table 1. HED and LED groups were equated on age, education, GPA, gender, family history of alcoholism, and intelligence. HED participants had higher scores on all alcohol-related variables compared to LEDs. They reported higher numbers of drinking days, more drinks per occasion, binge episodes and blackouts in the previous six months. HEDs also reported more alcoholism-related symptoms, higher alcohol cravings and more harmful consequences from drinking. They reported using alcohol as a coping strategy, to enhance their experience in social situations, and because they enjoyed the euphoria it caused. On average, HED participants started drinking around the age of sixteen, two years earlier than LEDs. In contrast, no differences between groups were detected in anxiety, depression, ADHD-like symptoms or personality measures of psychoticism, neuroticism and extraversion. However, HED individuals reported higher motor impulsivity, boredom susceptibility, and disinhibition. In addition, they rated the Stroop task as being more difficult than LEDs.

### 3.2. Task Performance

**Figure 2:**
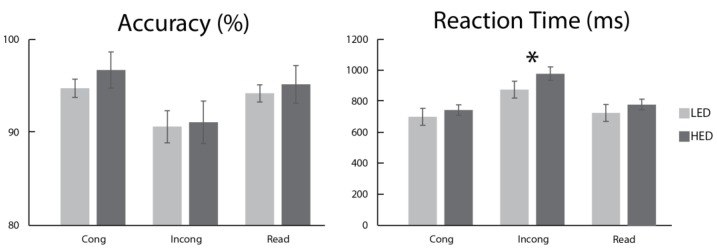
Lower accuracy and longer reaction times (RT) on INCONG trials confirm the Stroop interference effect. HED participants show longer RT time only on incongruent trials: * *p* < 0.05.

Accuracy and reaction times were analyzed with a mixed-model ANOVA with the factors of Group and Condition. As shown in Figure 2, there was a main effect of Condition on task accuracy (*F*_2,58_ = 27.04, *p* < 0.001) and reaction time (*F*_2,58_ = 269, *p* < 0.0001), confirming that the task successfully elicited the Stroop interference effect. The groups did not differ on accuracy (*F*_1,29_ = 0.22, *p* = 0.61) but overall accuracy was the lowest on the INCONG trials compared to both the CONG (*F*_1,30_ = 33.0, *p* < 0.0001) and READ (*F*_1,30_ = 27.38, *p* < 0.0001) trials. Similarly, reaction times (RT) were the longest on the INCONG trials (M = 923 ms), followed by READ (M = 753 ms) and CONG (M = 721 ms), with all conditions differing from each other (*p*’s < 0.0001). A significant Condition x Group interaction for RT (*F*_2,58_ = 5.44, *p* < 0.01) was driven by the HED individuals responding with significantly slower response times to INCONG stimuli (*F*_1,29_ = 4.22, *p* < 0.05) relative to LEDs (Figure 2). There were no significant task performance differences between men and women. Altogether, this indicates that regardless of gender HEDs were particularly sensitive to response conflict. Indeed, the interference effect, calculated as the RT difference between INCONG and CONG conditions, was positively correlated with all drinking measures (*p*’s < 0.05). Stroop task difficulty was positively correlated with binge frequency (r_s_ = 0.55, *p* = 0.002), average drinks per occasion (r_s_ = 0.43, *p* = 0.02, and the AUDIT (r_s_ = 0.46, *p* = 0.013).

### 3.3. Neuroimaging Results

**Figure 3:**
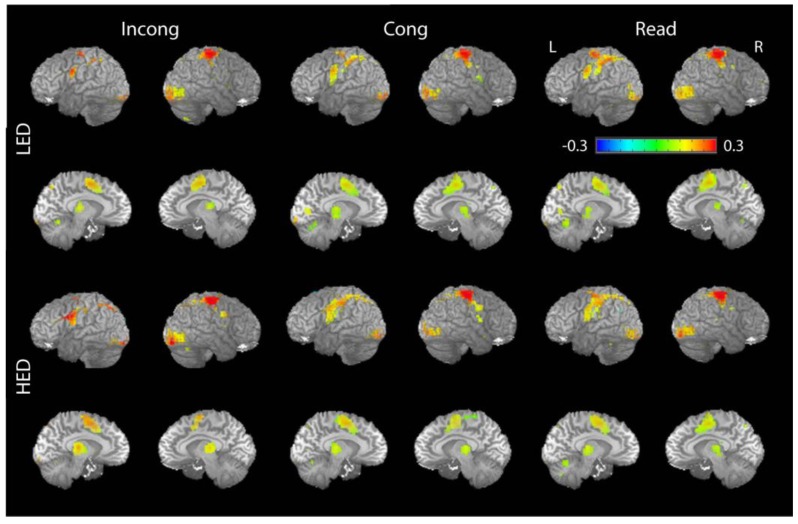
Lateral and medial views of group averaged activity for heavy (HED) and light episodic drinkers (LED) across task conditions at 4 s post stimulus. Voxel threshold was set at *p* = 0.001 with a false discovery rate (FDR) value of *q* = 0.003, *t* = 3.725. The color scale represents voxels with percent signal change coefficients between −0.3 and 0.3 that survive the threshold obtained from mixed-effects meta-analysis (MEMA).

**Figure 4:**
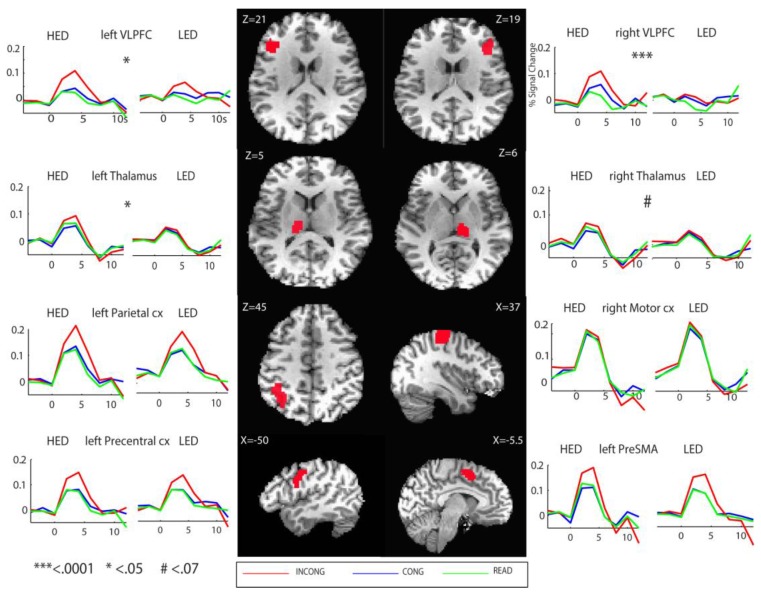
Group average time series expressed as % signal change of the blood oxygenated level dependent (BOLD) signal for the effects of Condition and Group. As shown in Table 2, greater BOLD contrast was observed in HEDs in ventrolateral prefrontal cortex (VLPFC) and the thalamus bilaterally. Response conflict evoked greater activity in the frontal and parietal association cortices overall, but only marginally in the thalamus and not in the sensorimotor cortex (*** <0.0001; * <0.05; # <0.07).

As shown in Figure 3, voxel-wise analysis of the peak activation showed a distributed activation pattern which is broadly consistent with previous studies. The task conditions activated inferior precentral, anterior ventrolateral prefrontal, sensorimotor, parietal, occipital cortices and the insula laterally, and the supplementary/presupplementary cortex and the thalamus medially. A robust main effect of Condition with stronger activation to INCONG compared to CONG and READ trials was observed across most association fronto-parietal activated areas but not in the precentral, sensorimotor, and visual cortices (Figure 4, Table 2). Extracted ROI time series were statistically assessed via mixed-model ANOVA and presented in percent signal change values (Figure 4, Table 2). The largest effects of Group were observed in the VLPFC and the thalamus bilaterally. INCONG trials elicited stronger activity in HED in both the VLPFC (*F*_1,29_ = 13.4, *p* < 0.001) and left thalamus (*F*_1,29_ = 5.3, *p* < 0.05) relative to LED (Figure 3 and Figure 4). Similar to the behavioral results there were no significant gender differences in the ROI analysis.

Nonparametric correlations of Spearman’s Rho were calculated between alcohol measures and extracted time courses for ROI peak activations across groups. Bilateral peak activation of the VLPFC to INCONG trials was positively correlated with binge episodes, blackouts, and the SMAST and AUDIT alcohol severity measures (*p*’s < 0.02). Peak activation of the left thalamus elicited by INCONG trials was positively correlated with binge episodes (r_s_ = 0.38, *p* = 0.035). There was a significant positive relationship between the rVLPFC and Stroop difficulty during both CONG (r_s_ = 0.43, *p* = 0.023) and INCONG (r_s_ = 0.466, *p* = 0.013) trials. Finally, when parsing apart Groups, HED individuals have a positive correlation between the rVLPFC with non-planning impulsivity (r_s_ = 0.601, *p* = 0.03) during INCONG trials.

## 4. Discussion

This study used fMRI to investigate behavioral and brain activity indices of cognitive control in heavy and light episodic drinkers during response conflict evoked by the Stroop task. As expected, the high conflict (INCONG) condition evoked greater activity in the fronto-parietal association cortices. Importantly, this activation was greater for HEDs in the VLPFC and thalamus bilaterally relative to LEDs. Conflict-induced activity in the bilateral VLPFC was positively correlated with levels of alcohol intake including binge episodes and the number of drinks consumed per occasion, as well as measures of alcoholism-related symptoms (AUDIT, SMAST) and blackouts. Reported binge episodes were further correlated with activity in the left thalamus. In the absence of accuracy deficits, HEDs had significantly slower RTs to INCONG stimuli, which presumably allowed them to maintain performance levels on par with LEDs. HEDs were particularly affected by response conflict as both the Stroop interference effect and task difficulty ratings were associated with alcohol intake measures. Taken together, these results suggest that heavy episodic drinkers benefit from engaging an expanded cognitive control network and responding more slowly to resolve stimulus conflict. These effects are especially pronounced in those with heavier drinking patterns and more binge episodes which may be indicative of the deleterious effects of excessive alcohol use.

Binge drinking participants in the present study were young, healthy individuals who showed no cognitive deficits on a standardized intelligence scale. Nonetheless, the observed group differences in brain activity are consistent with those found in AUD samples. Increased activity of the VLPFC has been reported on tasks probing working memory [38,39,101], response inhibition [35], and delay discounting [36]. Our results are most in line with the findings by Wilcox and colleagues [37] who used a multisensory Stroop and observed bilateral VLPFC hyperactivation and an overall increase in RTs in AUD participants compared to the control group. Despite a paucity of neuroimaging studies investigating cognitive control in binge drinkers, the results that have emerged are also similar to those found in AUD. Increased activity of the VLPFC has been reported on a task probing spatial interference [64] and declarative memory in young adult heavy drinkers [40]. A study of spatial working memory in adolescents reported greater activation in the right inferior frontal gyrus in male but not female adolescent binge drinkers [102]. Moreover, on the Eriksen flanker task, increased activity was induced by response conflict in the VLPFC under acute intoxication in social drinkers [103]. Inconsistent reports notwithstanding [33,62,104], accruing neuroimaging evidence indicates frontal hyperactivation in AUD and binge drinkers compared to low-drinking control groups. More studies are needed to corroborate these findings, but it appears that greater inferior frontal activation is elicited by tasks that impose higher demands on cognitive control by relying on deliberative functions and multidimensional contingencies [105].

This converging evidence is consistent with compensatory accounts of increased engagement across cognitive and emotional neurofunctional systems in alcoholism [21,23,106,107,108]. Extensive functional imaging evidence suggests that the VLPFC is activated by tasks probing cognitive control [8]. It has been proposed as a key area for inhibitory control of inappropriate motor responses [16,109] but it is also activated during attentional capture [110] and domain-general tasks that are cognitively demanding but that do not rely on inhibition [111,112]. Meta analyses have provided further insights into functional specificity within the VLPFC subregions [113]. However, it is increasingly clear that the VLPFC is an integral part of a network that is engaged by a range of tasks imposing attentional demands in the context of processing novelty, contingency monitoring and conflict resolution [110,111,114]. Functional connectivity studies have revealed extensive connections of the VLPFC with other parts of the lateral frontal cortex, the anterior cingulate, parietal, and temporal cortices [115]. Therefore, in response to increased demands imposed by multi-rule tasks, the VLPFC is likely to be recruited in a process of flexible network reconfiguration [114]. On that view, the brain functions as a dynamic, interactive system that handles changing environmental demands via targeted, yet flexible and integrated engagement of the relevant neurofunctional networks in order to optimize responding [116]. These networks interact across spatiotemporal scales, they are synchronized from local, specialized, to global-level networks and reflect typical [117] and pathological changes [118]. In the present study, greater activation of the VLPFC was elicited by response conflict selectively in HEDs and correlated with Stroop difficulty ratings. Furthermore, both of these variables were associated with various measures of alcohol intake and harmful drinking consequences. Therefore, the converging evidence suggests that in HEDs the task demands exceeded the normative network capacity, necessitating compensatory engagement of the VLPFC and adjustment of response strategy. The observed activation increase is associated with heavy alcohol intake and may reflect adaptation of the brain’s functional networks to the sequelae of heavy drinking. This interpretation is consistent with evidence indicating an expansion of primarily frontal networks in AUD. Muller-Oehring and colleagues [119] examined functional connectivity in sober alcoholics compared to matched controls during wakeful rest and reported expanded attention/salience network which comprised the inferior frontal cortex. The compensatory interpretation is supported by the correlation between better task performance and network enlargement [119]. Not all studies, however, show increased activity in brain regions implicated in cognitive control. Decreased activation has been reported in studies with simpler contingencies that rely on response inhibition such as the go/no go and stop signal tasks [31,32,33]. These tasks may not be challenging enough to generate high conflict and may not engage cognitive control at the level that would necessitate activation of additional areas [120]. The present results could serve as an indirect indication of impaired cognitive control in HED individuals.

Overwhelming evidence indicates that cognitive control is subserved by a distributed, but predominantly frontal, cortical network [4,6,13,121]. It has been established that AUD is associated with a range of impairments in the cognitive domain and neurophysiological changes in the frontal lobes [21,22,23,122]. This degradation of prefrontal functions results in impaired decision making and self-control which further contribute to the development of alcohol dependence [67,123,124,125]. The Stroop task probes top-down regulation by necessitating the suppression of prepotent responses in favor of controlled processing. The sensitivity of prefrontal regions to the effects of acute alcohol on response interference has been shown in previous imaging studies [17,19]. Results of the present study additionally indicate cognitive control deficits in young adult HEDs. This supports existing evidence that self-control impairments can contribute to excessive drinking [66,69,72]. Indeed, automatic modes of processing are associated with the increased risk of heavy alcohol use [126], its maintenance across time [127], and relapse in abstinent alcoholics [128].

Our study revealed group differences in thalamic activity with HEDs showing greater sensitivity to response interference. The increased activation was positively correlated with the number of binge episodes. Similar results were reported by Campanella and colleagues [65], with increased thalamic activity in HEDs during working memory in association with drinking levels. In alcohol-dependent participants, increased thalamic activity was observed during an auditory go/nogo [35] and multisensory Stroop task [37], but lower activity during working memory [129]. Indeed, there is growing evidence that the thalamus plays a modulatory role in integrating activity across different levels of the neuraxis and that it contributes to cognitive control and flexible action selection [130,131,132,133,134]. In the present study, HED participants not only exhibited greater thalamic activity, but they also rated the task as being significantly more difficult and responded with longer RTs than the LED subjects indicating that the task imposed increased cognitive demands. It is likely that the increased difficulty was accompanied by heightened arousal [135,136] which is in part subserved by the thalamus [137,138]. Another possibility is that the increased activation of the thalamus reflects neuroadaptive changes resulting from frequent bouts of heavy drinking [42,45]. The thalamus is sensitive to long-term excessive alcohol use and is implicated in a range of deficits across distributed neural circuits [139]. Smaller thalamic volumes are predictive of subsequent relapse and alcohol intake in chronic alcoholics [140]. Thalamic hyperactivity could, therefore, reflect its sensitivity to protracted heavy use and its compensatory engagement during increased response conflict difficulty [106].

Measures of impulsivity and disinhibition were positively correlated with alcohol intake variables in the present study, confirming well-established associations between AUD and traits of impulsivity, hyperactivity, and sensation/novelty seeking [141,142,143,144]. Dysregulation of impulse control underlies the inability to maintain inhibitory control over drinking, which has been considered fundamental to addiction [67,123,145,146,147]. Findings suggest that the vulnerability to alcoholism shares a common genetic component with a cluster of impulsivity traits that may predispose individuals to AUD [144,148,149]. Recent evidence converges on dopaminergic modulation of impulsive behavior [150,151], suggesting that the same genetic pathways may mediate both addiction and impulsivity [152,153]. Overall, these findings are strongly suggestive of shared genetic vulnerability to AUD and externalizing traits. The present study cannot speak to the issue of possible pre-existing characteristics of the HED sample unrelated to drinking levels that may have resulted in the observed group differences. However, the behavioral and brain activity findings are correlated with a range of alcohol intake measures.

Moreover, findings across different studies indicate that the compensatory activity increase may reflect deficits as a function of alcohol-induced neurotoxicity. In a working memory task, Campanella and colleagues [65] reported positive correlation between alcohol consumption and activity in the dorsomedial prefrontal cortex in binge drinkers. Wetherill and colleagues [154] tracked the development of alcohol habits in young adults and similarly reported a positive relationship between alcohol-induced blackouts and increased prefrontal on a response inhibition task. In a study of alcohol-dependent participants, lifetime alcohol consumption was predictive of activity in the posterior cingulate cortex and midbrain during a multisensory Stroop task [155]. Taken together, the evidence suggests that the disinhibitory traits are implicated in drinking initiation and maintenance via impaired self-control, but that the group differences in neural function may at least in part reflect alcohol-induced neurotoxicity.

The development of addiction is an exceedingly complex process mediated by environmental risk factors, and interindividual variability [156] due in part to a person’s genetic makeup [52,157,158,159,160]. However, heavy episodic drinking is associated with deficits in neural functioning in response to conflict-inducing situations with increased vulnerability in adolescents and emerging adults [48,57,161]. These neural compromises are often not detectable by behavioral measures but can be revealed by measures of neural function [74,162,163] and may signify early trajectory toward compulsive intake characterizing AUD [42,44,164]. Given that individuals who engage in most hazardous binge drinking are young and vulnerable to neurotoxicity, it is of paramount importance to better understand the neural indices associated with excessive drinking. Such insights may inform development of therapeutic, personally tailored approaches and prevention strategies.

## Figures and Tables

**Table 1:** Subjects characteristics (mean ± SD or *n* (%)) for heavy (HED) and light episodic drinking (LED) groups.

	HED (*n* = 14)	LED (*n* = 17)	Stat. Value (U/chi ^a^)	*p*
% Female	64.3%	52.9%	0.406 ^a^	ns ^a^
Age	23.8 ± 3.4	25.5 ± 4.1	90.5	ns
Family History Positive	50%	58.8%	0.241 ^a^	ns ^a^
Education Years	15.1 ± 1.9	15.9 ± 2.3	81	ns
Undergraduate Grade Point Avg.	3.40 ± 0.38	3.59 ± 0.33	79	ns
Stroop Task Difficulty Ratings	3.07 ± 0.62	2.00 ± 0.78	32	0.001
In the past 6 months				
No. of drinking days/wk	2.67 ± 1.2	1.35 ± 0.96	46	0.003
No. of drinks/occasion	5.25 ± 2.6	2.2 ± 1.2	34	0.001
No. of binge episodes	15.21 ± 15.2	0.41 ± 0.7	0.000	<0.001
No. of alcohol-induced blackouts	3.14 ± 2.9	0.18 ± 0.5	33	<0.001
Max No. of drinks in 24 h	12.57 ± 9.8	3.41 ± 2.06	11	<0.001
Age Onset of Alcohol Use	15.72 ± 1.49	18.13 ± 2.13	31.5	0.007
Severity of Alcoholism (SMAST)	2.86 ± 2.4	0.76 ± 1.0	47	0.003
Alc. Use Disorder Ident. Test (AUDIT)	13.43 ± 5.80	3.82 ± 1.55	4.5	<0.001
Motivation				
Enhancement	2.33 ± 0.333	1.69 ± 0.46	26.5	<0.001
Social	2.62 ± 0.405	2.04 ± 0.44	40.5	0.002
Conformity	1.41 ± 0.564	1.43 ± 0.45	104	ns
Coping	1.67 ± 0.472	1.19 ± 0.29	37.5	0.002
Drinking Consequences (B-YAACQ)	8.86 ± 6.11	2.76 ± 3.09	47	0.004
Alcohol Craving (PACS)	7.71 ± 4.53	2.53 ± 2.27	36	0.001
Anxiety (GAD-7)	2.07 ± 1.68	3.06 ± 2.63	100	ns
Depression (PHQ-9)	3.07 ± 2.70	3.18 ± 2.96	118	ns
ADHD Symptomology (ASRS)	1.64 ± 1.59	1.06 ± 1.34	94.5	ns
Impulsivity				
Attention	2.06 ± 0.478	1.83 ± 0.39	75	ns
Motor	2.23 ± 0.632	1.78 ± 0.42	61	0.036
Non-Planning	2.09 ± 0.739	1.78 ± 0.48	84.5	ns
Sensation Seeking				
Experience	4.23 ± 0.806	3.97 ± 0.86	90	ns
Boredom	4.11 ± 0.650	3.59 ± 0.76	65.5	0.05
Thrill	3.69 ± 1.15	3.41 ± 1.28	97.5	ns
Disinhibition	3.81 ± 0.722	2.94 ± 1.03	54.5	0.018
WASI-II Percentile	69.36 ± 21.11	72.81 ± 21.40	99.5	ns
Eysenck Personality				
Neuroticism	3.08 ± 2.29	3.71 ± 3.75	110	ns
Psychoticism	2.38 ± 2.26	2.18 ± 1.38	110	ns
Extraversion	9.46 ± 2.63	7.71 ± 3.57	80.5	ns

^a^ Tested with Chi-Square; all other comparisons performed with the nonparametric Mann–Whitney U-test. SMAST: Short Michigan Alcoholism Screening Test; AUDIT: Alcohol Use Disorder Identification Test; B-YAACQ: Brief Young Adult Alcohol Consequences Questionnaire; PACS: The Penn Alcohol Craving Scale; GAD7: Generalized Anxiety Disorder; EPQ: Patient Health Questionnaire; ASRS: Adult ADHD Self-Report Scale; WASI-II: Wechsler Abbreviated Scale of Intelligence.

**Table 2:** Statistical results for regions of interest (ROI) and their Talairach coordinates, trial condition effects, and INCONG vs. CONG & READ (INC > C + R) results for HEDs and LEDs.

Area	Tal. Coord (LPI)	m.e.Group (*F*_2,58_)	m.e.Cond (*F*_2,58_)	Cond x Group	HED: INC > C + R (*F*_1,13_)	LED: INC > C + R (*F*_1,16_)
L. VLPFC	−40.1, 25.8, 21.3	5.3 < 0.05	56.3 < 0.0001	ns	17.6 < 0.001	116.2 < 0.001
L. Thalamus	−11.6, 20.6, 5.2	5.9 < 0.05	14.6 < 0.001	ns	11.2 < 0.006	ns
L. PreSMA	−5.5, 4.7, 49.2	ns	63.9 < 0.0001	ns	29.4 < 0.001	35.6 < 0.0001
L. PreCent. cx	−50.6, 0.6, 31	ns	48.3 < 0.0001	ns	17.2 < 0.001	36.6 < 0.0001
L. Parietal cx	−37.5, −49.3, 45.5	ns	43.1 < 0.0001	ns	23.9 < 0.001	18.8 < 0.001
L. Insula	−28.7, 16.6, 9.9	ns	30.2 < 0.0001	ns	9.2 < 0.01	26.3 < 0.0001
R. VLPFC	42.1, 28.7, 19.4	17.7 < 0.0003	32.4 < 0.0001	4.4 < 0.05	17.7 < 0.001	12.1 < 0.01
R. Thalamus	12.6, −22.2, 6.1	3.6 < 0.07	5.9 < 0.05	ns	ns	ns
R. Insula	40.0, 5.4, 11.2	ns	6.9 < 0.05	ns	ns	5.86 < 0.05
R. PreCent. cx	37.3, −26.4, 54.5	ns	ns	ns	ns	ns
R. Motor cx	37.1, −21, 56.1	ns	ns	ns	ns	ns
